# Renewing Lost Genetic Variability with a Classical Yeast Genetics Approach

**DOI:** 10.3390/jof9020264

**Published:** 2023-02-16

**Authors:** Ameya Pankaj Gupte, Debora Casagrande Pierantoni, Angela Conti, Leonardo Donati, Marina Basaglia, Sergio Casella, Lorenzo Favaro, Laura Corte, Gianluigi Cardinali

**Affiliations:** 1 Department of Agronomy Food natural Resources Animals and Environment (DAFNAE), University of Padova, 35020 Legnaro, Italy; 2 Department of Pharmaceutical Sciences, University of Perugia, 06121 Perugia, Italy

**Keywords:** yeast classical genetics, metabolomic fingerprint, sporulation, recombination, stress, glucose, formic acid, copper, FTIR

## Abstract

Due to their long domestication time course, many industrial *Saccharomyces cerevisiae* strains are adopted in numerous processes mostly for historical reasons instead of scientific and technological needs. As such, there is still significant room for improvement for industrial yeast strains relying on yeast biodiversity. This paper strives to regenerate biodiversity with the innovative application of classic genetic methods to already available yeast strains. Extensive sporulation was indeed applied to three different yeast strains, specifically selected for their different origins as well as backgrounds, with the aim of clarifying how new variability was generated. A novel and easy method to obtain mono-spore colonies was specifically developed, and, to reveal the extent of the generated variability, no selection after sporulation was introduced. The obtained progenies were then tested for their growth in defined mediums with high stressor levels. A considerable and strain-specific increase in both phenotypic and metabolomic variability was assessed, and a few mono-spore colonies were found to be of great interest for their future exploitation in selected industrial processes.

## 1. Introduction

Apart from being a powerful model system to answer hallmark biological questions, the species *Saccharomyces cerevisiae* plays a key role in many industrial applications [1,2]. The domestication of *S. cerevisiae* strains independently occurred in many processes even before the first microbes were observed [3,4,5]. After centuries of continuous growth under favorable conditions, with nutrients readily and abundantly available, many domesticated *S. cerevisiae* strains have partly or entirely lost the ability to reproduce sexually [6], gotten more and more tolerant to specific stressors frequently faced in industrial plants, and metabolized a few sugars more rapidly than natural strains [7]. This is reminiscent of the so-called “domestication syndrome,” already described in 1868 by Darwin, where organisms under domestication tend to drop undesirable and/or unselected traits and acquire attributes that make them successful in human-shaped environments [8,9].

In the past centuries, brewers, bakers, and, to some extent, even winemakers were used to perform subsequent fermentations using yeast strains from an old batch [5,10]. The back-sloping procedure was indeed essential to maintain the stability of the final product, thus ensuring the economic sustainability of the process of interest. Both refrigeration and the advent of pure cultures to start the fermentation further enhanced the stability of the final industrial and/or artisanal products [5,11]. Paradoxically, as soon as bakers and brewers recognized the pivotal role of *S. cerevisiae* strains in the fermentation and began to isolate pure cultures, the yeast genetic diversity severely decreased as pure cultures were more and more adopted and clonal batches were maintained by refrigeration [12]. As such, many yeast strains industrially used today, primarily those adopted in bioethanol, wine, and beer fermentations, are often utilized mostly for historical reasons rather than scientific ones [3,13,14,15]. Furthermore, since the demand of customers as well as industries has turned and continued to turn, there is still significant scope for improvement of industrial strains despite their long domestication time course. The non-genetically modified organisms (non-GMO) approaches, particularly for food and beverage yeast applications, should be considered the most since they do not suffer from any issues with consumer acceptance and/or specific legislation [16,17].

**Figure 1:**
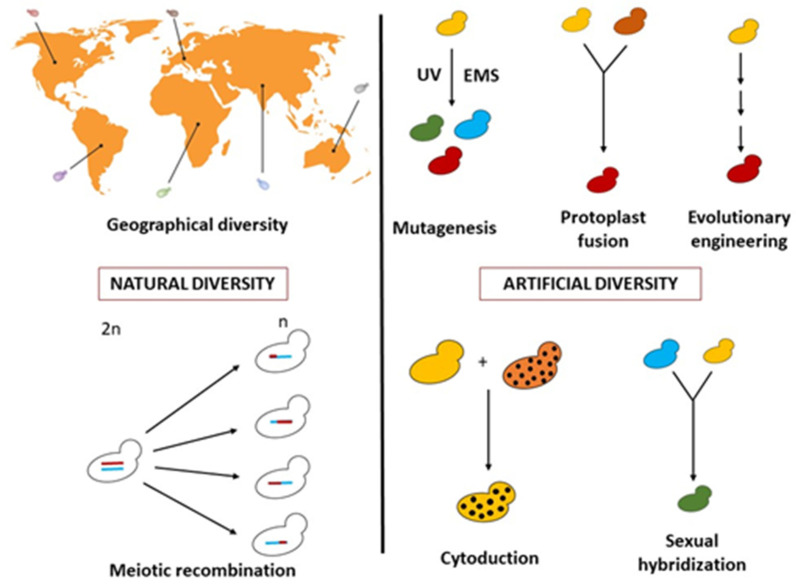
Methods for obtaining genetic variability in yeast adapted from Steensels et al. [18] (UV ultraviolet, EMS, ethyl methane sulfonate).

There are multiple non-GMO strategies to provide suitable yeast strains for specific industrial goals (Figure 1), as elegantly reviewed [18]. A very powerful approach is to look for natural biodiversity by selecting a yeast able to operate best in a specific industrial process [13,18]. Indeed, recent metagenomic surveys underpin the fact that the natural yeast biodiversity is immense and largely unexplored, with the existing industrial strains corresponding to only a small share of the natural biodiversity [19,20,21]. An alternative route is to regenerate biodiversity with the innovative application of classic genetic methods to already available yeast strains [18]. Both the search for natural biodiversity and the regeneration aim at selecting the best phenotypes.

This work specifically focused on the latter strategy, choosing *S. cerevisiae* as a yeast candidate with a long biotechnological history as well as being a model organism with a homothallic nature. In contrast to higher eukaryotes, yeast gametes enter a haploid life cycle that is substantially similar to the diploid mitosis-based cycle. By taking advantage of this feature, it is possible to produce recombination of important traits by extensive sporulation, obtaining as many combinations as the spores. As an example, *Drosophila* was suggested as a possible niche for sporulation and mating as, different strains of *Schizosaccharomyces japonicus* isolated from Drosophila showed variation for pheromone-related genes [22]. Various sporal cultures can be directly tested or induced to undergo homothallic switching and subsequent diploidization. The output of this route is a collection of diploid cultures that are homozygous at all loci since they are derived from the conjugation of genetically identical cells. These cultures are theoretically very stable, since mutations would rarely affect the phenotype due to the very low, if any, heterozygosity and would provide the most extreme effects of quantitative trait loci. Whether the genome renewal [23] could reintroduce heterozygosity and to what extent is a matter of obvious importance at both the theoretical and practical levels [24].

This work applied extensive sporulation to three different yeast strains, precisely selected for their different origins as well as backgrounds, with the aim of clarifying how new variability was generated from sporulation. Three different hypotheses were investigated: (i) the isolation of diploid homozygous mono-spore colonies (MSCs) allows to verify the amount of variability produced in the sporulation of each parental genotype, (ii) the quantitative traits analyzed show significant differences from the parental strains; and (iii) the obtained variability strictly depends on the starting parental genotype under sporulation. A new method for obtaining MSC without the use of a micromanipulator has been specifically developed, avoiding the introduction of any form of selection to exclusively focus on the extent of variability generated by recombination under sporification. For this purpose, mono-spore colonies (MSCs) were indeed randomly chosen and sequentially analyzed with increasingly informative tests also considering the presence of specific stressors (i.e., high levels of glucose or formic acid, or copper sulphate).

## 2. Materials and Methods

### 2.1. Yeast Strains and Growth Conditions

Three *S. cerevisiae* strains with different backgrounds and geographical origins were specifically selected for this study (Table 1). Strains were maintained in 20% glycerol stocks at −80 °C and usually plated on YPD agar (Yeast extract-10 g L^−1^, Peptone-20 g L^−1^, Glucose-20 g L^−1^, Agar-15 g L^−1^) and incubated at 30 °C for 48 h. Screening for sporulation was performed at 30 °C for 7–21 days on SM1 (potassium acetate-10 g L^−1^), McClary’s Acetate medium (sodium acetate-8.2 g L^−1^, glucose-1 g L^−1^, yeast extract-2.5 g L^−1^, potassium chloride-1.8 g L^−1^, Agar-15 g L^−1^) and modified sporulation medium (MSM) (potassium acetate-10 g L^−1^, yeast extract-0.5 g L^−1^, glucose-1 g L^−1^, Agar-15 g L^−1^) [25]. All the media were sterilized by autoclaving at 121 °C for 20 min before plating.

*S. cerevisiae* TC1517 has been isolated from grape marcs [26] and has shown great promise in terms of fermenting abilities. *S. cerevisiae* YI30 was chosen as a strong candidate for lignocellulosic ethanol because of its high inhibitor and temperature tolerance [27]. The Canadian strain *S. cerevisiae* YVGC13A was chosen to evaluate the variability of a strain directly isolated from vine bark, which is currently considered the main natural reservoir of *S. cerevisiae* strains that could participate in alcoholic fermentation [28].

In addition, stressing experiments and metabolomic studies using Fourier-Transform Infrared Spectroscopy (FTIR), were carried out by inoculating yeast cultures at OD_600_ = 0.1 in 100 mL of filter sterilized (0.22 µm) synthetic defined (SD) medium containing 6.7 g L^−1^ of Yeast Nitrogen Base medium (YNB, Difco Laboratories, Detroit, MI, USA) and 20 g L^−1^ of glucose and grown them for 16–18 h at 30 °C under shaking at 120 rpm.

### 2.2. Spore Production and Sporulation Efficiency

A fresh single colony of each strain was inoculated into 5 mL YPD broth, and microaerophilic conditions were maintained while shaking at 30 °C for 16 h. The suspension was centrifuged at 3000 rpm for 5 min and the obtained pellet was washed twice with a sterile 9 g L^−1^ NaCl solution. Washed yeast cells were resuspended in 0.5 mL saline solution, and aliquots of 100 µL were plated on MSM. Plates were then incubated at 30 °C for 7–21 days. To avoid moisture loss, plates were sealed with Parafilm^®^ (Bemis Company, Inc., Neenah, WI, USA). Microscopic observation was performed every week to observe spore development. The spores were counted after the addition of methylene blue (MB) to the spore suspension to allow the distinction of the living cells from the dead ones that were excluded. The number of dyads, triads, and tetrads was counted using a counting chamber (Thoma, Germany). Sporulation efficiency, a measurement of the amount of cells that undergo sporulation, was calculated by microscopic observation of the sum of triads and tetrads divided by the total asci. Sporulation efficiency (SE) [29] was then calculated as follows:

$$\%\;\mathrm{Sporulation}\;\mathrm{efficiency}=\frac{\mathrm{Number}\;\mathrm{of}\;\mathrm{triads}+\mathrm{Number}\;\mathrm{of}\;\mathrm{tetrads}}{\mathrm{Number}\;\mathrm{of}\;\mathrm{total}\;\mathrm{spores}}\times 100$$

### 2.3. Screening of Temperature Tolerance of PS and Spores

In order to develop a quick method to produce MSCs, the minimum temperature required to kill vegetative cells of each parental strain (PS) within a population of spores was screened. Each strain was grown in YPD broth for 16 h at 30 °C and centrifuged at 3000 rpm for 5 min. Cells were suspended in sterile saline to a final density of 1 × 10^7^ cells mL^−1^, and 0.5 mL of cell suspension was transferred to a sterile 1.5 mL tube and exposed for 10 min at different temperatures from 55 to 67 °C at 2 °C intervals.

Each treated suspension was observed microscopically using the MB viability assay [30], and a proper dilution was plated on YPD agar plates in triplicate. The asci of yeast were broken using zymolyase treatment, as explained in Section 2.4 [31], and the related spores’ sensitivity was tested at 63, 65, and 67 °C. The quantification protocol was the same as for vegetative cells.

### 2.4. Production of Mono-Spore Colonies

The ascospore isolation method described by Bahalul et al. [31] was modified to avoid the use of diethyl ether. Briefly, colonies grown on MSM agar were scraped and resuspended in sterile, demineralized water. This high-density suspension of asci was heat-treated at 65 °C for 10 min to kill vegetative cells and then processed with zymolyase treatment (Zymolyase^®^-100T, ICN; 100 U mL^−1^ in 1M sorbitol) by extending the incubation time to 1 h. Sterile glass beads (400–600 µm) were used to apply shear force on ascus walls. The resultant spore suspension was observed microscopically using MB to check for the presence of asci or viable vegetative cells. Each suspension was then properly diluted and plated on YPD agar plates supplemented with 5% (*w*/*v*) glucose. Thus, obtained colonies were referred to as MSCs. Up to 100 MSCs of each PS were stored in 20% glycerol stock at −80 °C. All MSCs were then grown on YPD 5%, transferred to MSM, and incubated at 30 °C for 7–21 days to test their sporulation ability. Microscopic observation was used to check the occurrence of asci and confirm the homothallic phenotype of the parental strains.

### 2.5. Phenotypic Variation in MSCs

Thirty MSCs were randomly selected from each PS and grown in SD broth at 30 °C for 16 h. These pre-cultures were inoculated in the same broth to obtain a final OD_600_ of 0.1 in a final volume of 200 µL. The experiment was run in 96-well plates in triplicate (TECAN Spark^®^ 10M, Salzburg, Austria) at 30 °C (flat-bottom cell culture plate with instrument lid; interval time-5min; shaking-60 s; shaking mode-orbital; amplitude-2.5 mm). Growth curves were plotted using the *Pyphe-growthcurves* tool. Growth parameters such as maximum growth rate (max_slope), time at max_slope (t_max), and lag phase (Lag) were obtained with the same tool [32]. The definition of the growth parameters given by *Pyphe-growthcurves* are as follows: max_slope-maximum slope of growth curve, t_max-time at which maximum growth slope of curve is reached, lag-lag phase.

Principal component analysis (PCA) was performed [33] considering these growth parameters, and the principal component scores and loading vectors were combined in a biplot used for the selection of specific MSCs for further studies. Additionally, a Student’s *t*-test was performed to determine if the observed differences were statistically significant. Moreover, at least one MSC with growth parameters similar to those of the parents was also included.

The resulting selected 12 MSCs and their parental strain were then grown under specific stressungconditions to observe growth parameters and, as reported in Section 2.7, metabolomic changes at different stressunglevels. The MSCs and PS were pre-inoculated in SD medium and grown at 30 °C for 16 h. Each PS and respective MCS were inoculated (OD_600_ = 0.1) in SD medium with no stressing agent and in the presence of low and high concentrations of the stressing agent. Each test was performed in triplicate at 30 °C in a 96-well microtiter plate (TECAN Spark^®^ 10M, Austria) with the same protocol described above.

### 2.6. Metabolomic Fingerprint at the End of Growth

Cell suspensions, prepared as detailed in Section 2.1, were centrifuged (4500× *g*, 5 min), washed twice with distilled sterile water, and re-suspended in 5 mL HPLC (High-Performance Liquid Chromatography) grade water to the final concentration of OD_600_ = 12. From each culture, 105 µL volume were sampled for three independent FTIR readings (35 µL each, according to the technique suggested by Essendoubi and colleagues [34].

### 2.7. Metabolomic Fingerprint under Stress

The FTIR analysis was also applied to investigate the metabolomic response under the stress of the selected MSCs cultures compared to their respective parental strains. MSCs and parental strain cultures were grown under different concentrations of stressing agents, as detailed in Table 1. However, yeast cultures were pre-inoculated at OD_600_ = 0.1 in 15 mL tubes with 7 mL of SD medium and grown at 30 °C under shaking at 120 rpm. Cell growth was stopped after 15 h. Each cell suspension was adjusted to an OD_600_ = 0.2 in a 2× fresh SD medium. A total of 100 µL of each standardized cell suspension was seeded in each selected well of a flat-bottom 96-well microtiter plate and brought to the final volume of 200 µL by adding 100 μL of a 2× solution of the respective stressing agent. Control (0% stressor concentration) was obtained by re-suspending cells in sterile, distilled water. All tests were carried out in triplicate. The growth was monitored in the TECAN as described above. The samples were collected at the end of the log phase of growth and processed for FTIR analysis [34].

### 2.8. FTIR Data Analysis

FTIR spectra were recovered from the OPUS software version 6.5 (Bruker Optics GmbH, Ettlingen, Germany) and transferred to MS Excel. Principal Component Analysis (PCA) and Significant Wavelengths Analysis (SWA) were performed in an R environment. SWA was employed to select the FTIR spectral regions with statistically significant differences in the comparison between the spectra of parental and MSCs cultures from the different experimental conditions tested [35]. In addition, pairs of spectra, each with three replicates, were compared using the Student’s *t*-test for each wavelength separately. For each wave number, the calculated *p*-value was recorded. Significant wavelengths were selected based on *p* < 0.01. Hierarchical cluster analysis was performed with MetaboAnalyst 5.0 [36]. Data were filtered based on interquartile range, normalized to the sample median, and scaled by Pareto scaling. Hierarchical cluster analysis (HCA) was employed to highlight the metabolic differences under stress between MSCs and PS cultures, using the Euclidean correlation method and the ward.D clustering algorithm. Significant wavelengths were selected based on these criteria: *t*-test (*p* adjusted < 0.05) and one-way ANOVA (*p*-value < 0.05).

## 3. Results and Discussion

### 3.1. Efficiency of Sporulation (SE) and Development of an Easy and Effective Protocol for MSCs Production

The three strains of *S. cerevisiae* were specifically selected for their different geographical origins and phenotypic backgrounds (Table 1). To develop a simple yet efficient protocol for obtaining high numbers of MSCs, the parental strains were first tested for their sporulation efficiency (SE) once plated on different media [25,37,38]. The highest SE was obtained on MSM plates, confirming literature data on the role of nutrient deficiency and non-fermentable carbon sources, such as acetate, in inducing sporulation [39] and on the involvement of the salt acetate cation in promoting the SE of yeast strains [38]. Sporulation media was indeed modified by Petersen et al. [40] to increase the SE. The addition of yeast extract to MSM also improved the SE, as reported by Tremaine et al. [41]. Notably, YI30 showed the highest SE (85.5%), and the other two yeast strains displayed slightly lower values (*S. cerevisiae* TC1517 and YVGC13A, at 68.4, and 64.1% SE, respectively).

Dawes and Hardie proved that vapors of diethyl ether in an agar plate or ether in liquid media kill the vegetative yeast cells, keeping spores alive [42]. This was previously applied once a protocol combining glusulase treatment, sonication, and separation of hydrophobic spores using diethyl ether was developed [43]. The diethyl ether protocol was also adopted after clubbing it with zymolyase and microbead treatment [31].

In the present study, temperature rather than diethyl ether was employed to kill vegetative cells [31], thus avoiding the use of an extremely flammable and volatile chemical solvent. Once exposed to high temperatures, the ascospores displayed greater tolerance than the respective vegetative cells. Separate experiments showed that vegetative cells tolerated temperatures up to 63 ± 0.5 °C. When the temperature was increased to 65 ± 0.5 °C, the parental strain was unable to grow, but the ascospores were able to produce colonies on YPD agar medium. These results are in line with those of Rachon et al. [44], who already observed a significant difference in temperature tolerance for vegetative cells and ascospores at 65 °C.

In order to optimize the ascospore separation protocol step, sporulated parental strains were scraped from MSM agar plates and suspended in sterile demineralized water. The zymolyase treatment, developed by Bahalul et al. [31], was found to be efficient with the extension of the zymolyase treatment to one hour. The combination of zymolyase and glass beads treatment was crucial to separate ascospores from broken asci, followed by heat treatment at 65 °C for 10 min.

Microscopic observation showed that around 60% of the asci were disrupted, releasing circular and refractive spores in suspension. These suspensions gave rise to individual colonies, referred to as MSCs, once plated on YPD agar medium.

Around 100 MSCs from each parental strain were thus obtained, and their homothallic nature was investigated as detailed in the Section 2.4. All parental strains were confirmed to be phenotypically homothallic, since all MSCs tested were able to produce spores.

### 3.2. Growth of MSCs from Each Parental Strain in SD Broth

**Figure 2:**
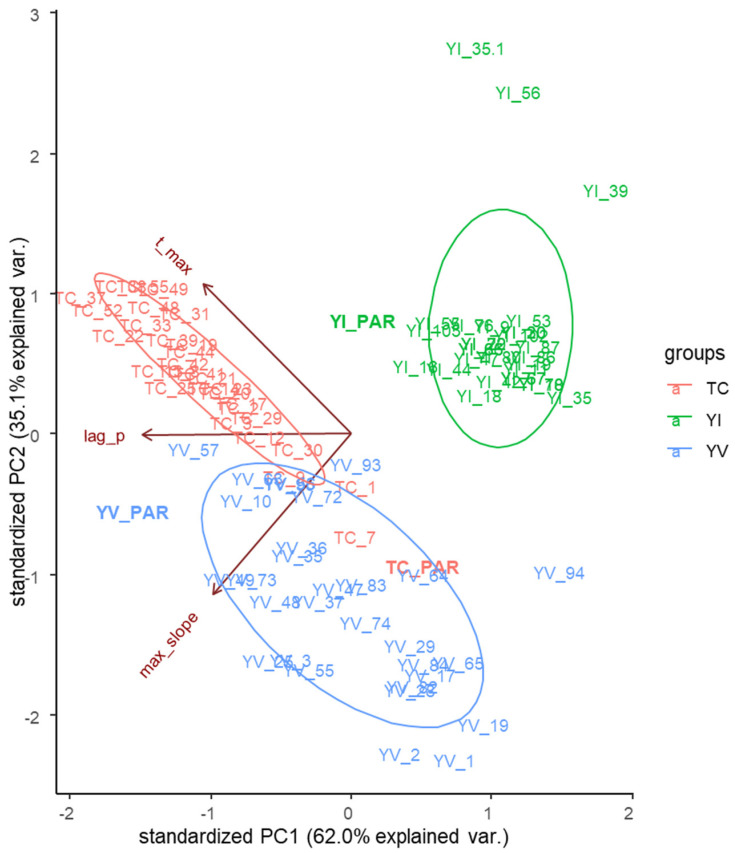
PCA biplot obtained from the growth parameters of all the 90 MSCs selected for the study. Input variables: Lag, t_max and max_slope growth parameters obtained using the *pyphe-growthcurves* tool from the growth curves of YVGC13A (YV, blue), TC1517 (TC, red), and YI30 (YI, green) cultures in SD with 2% glucose.

Thirty randomly selected MSCs from each parental strain were first screened for their growth at 30 °C in SD medium with 2% glucose. OD_600_ was monitored for 24 h at 30 °C using a 96 well plate reader (TECAN Spark^®^ 10M, Austria). The generated growth curves were processed using the *pyphe-growthcurves* tool to assess specific parameters such as max_slope, t_max, and lag used for the PCA analysis of Figure 2.

The first two principal components explained 97% of the variance between all the MSCs cultures (PC1: 62% and PC2: 35% of the variance). The spatial distribution of the MSCs cultures indicated a clear signature of the respective parental strains, suggesting that both ecological origin and geographical background are of great importance for the phenotypic variation triggered by sporulation. Camarasa et al. [45] observed similar results when metabolic traits were considered as differentiating parameters to understand the origin of *S. cerevisiae* strains. Interestingly, the growth performances of each parental strain remained outside the confidence ellipse, indicating higher variation between the growth of the parental strain and that of the corresponding MSCs. The highest variability was found within the monosporal progeny of the environmental yeast YVGC13A. Conversely, most of the MSCs from the YI30 and TC1517 strains formed a compact group, except for a few MSCs positioned outside the confidence ellipse.

According to PC1, the Lag Phase parameter was the most differentiating between groups. The other two parameters mainly contributed to the separation of the YVGC13A cluster from those of YI30 and TC1517 along the PC2.

**Figure 3:**
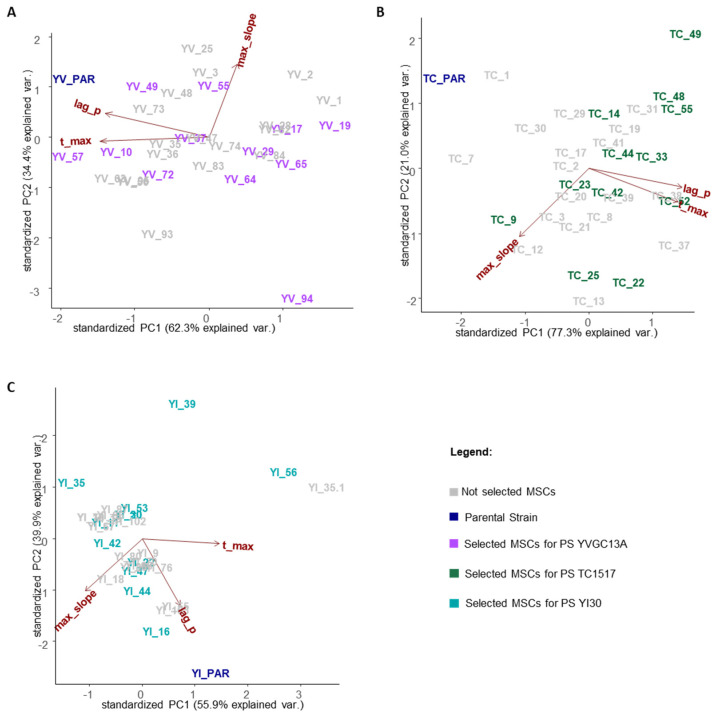
PCA biplot of growth parameters obtained from the 30 MSCs selected from the sporulation of each *S. cerevisiae* parental strain. Input variables: Lag, t_max and max_slope growth parameters obtained using the *pyphe-growthcurves* tool from for 30 MSCs of the parental *S. cerevisiae* strain YVGC13A (**A**), TC1517 (**B**) and YI30 (**C**). Parental strains are reported in blue, not selected MSCs cultures in grey and the twelve MSCs cultures selected for the next step of the analysis in violet (**A**), green (**B**) and light blue (**C**).

The same analysis was then carried out separately for each tested progeny (Figure 3). In all cases, most of the variance is distributed along the PC1, specifically 62.3, 77.3, and 55.9% for the YVGC13A, TC1517, and YI30 strains, respectively.

Interestingly, as already underlined in Figure 2, the parental strain was not part of the distribution of the variance of the relative MSCs cultures. Moreover, the three parameters differentially shaped the variance within each population, with lag and t_max as the main drivers for PC1 in the YVGC13A and TC1517 populations, while separately contributing along both PC1 and PC2 for the YI30 MSCs cultures.

Overall, these data already suggest that sporulation triggered phenotypic differences during aerobic growth in the presence of glucose. To further assess this evidence, twelve out of the 30 MSCs tested in each group were selected to undergo FTIR fingerprinting. MSCs were selected according to their statistically different growth parameters (*p*-value < 0.01) with respect to their parental strain (Tables S1–S3). Moreover, at least one MSCs with growth parameters such as those of the parental strain was included in the shortlist.

### 3.3. Metabolomic Fingerprinting of Selected MSCs

**Figure 4:**
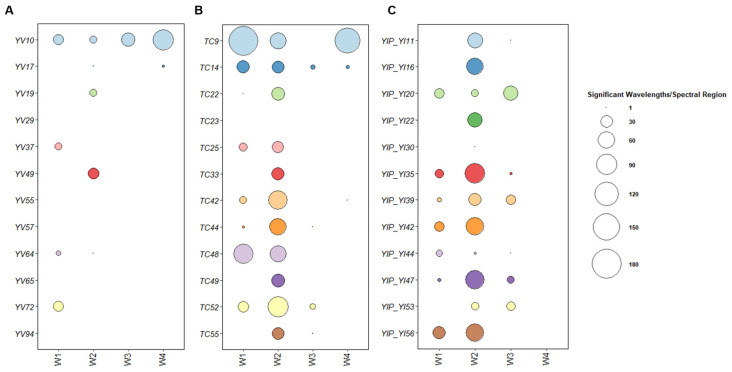
Number of significant different wavelengths detected in the comparison between each *S. cerevisiae* parental strain and the selected 12 MSCs. Spectra were compared using the Student’s *t*-test for each wavelength separately. The number of wavelengths with statistically significant difference (*p* < 0.01) was calculated for each specific spectral area separately, namely: fatty acids (W1), amides (W2), mixed region (W3) and carbohydrates (W4) regions. (**A**–**C**): MSCs of YVGC13A, TC1517 and YI30, respectively.

The selected MSCs were grown in SD broth supplemented with 2% glucose, and the cells were harvested at the end of the exponential phase to analyze the metabolomic fingerprint of their primary metabolism. The “R” script for Significant Wavelengths Analysis (SWA) was then adopted to compare all the statistically relevant differences between the spectra of PS and each related MSC [35]. Significant wavelengths were selected based on the Student’s *t*-test (*p* < 0.01, and their number was computed within each spectral region (Figure 4).

The FTIR fingerprints of monosporal cultures from the parental strain YVGC13A (Table S4, Figure S1) showed little to no variability, except for the MSC YV_10, which displayed significant differences in all the spectral regions tested. Notably, the highest variation was observed in the carbohydrate region of the FTIR spectrum (Figure 4A). Although the five MSCs YV_29, YV_55, YV_57, YV_65, and YV_94 showed significantly different growth kinetics from their parental cells (Table S1), these differences did not induce significant changes in their metabolome.

On the contrary, higher variation of the metabolomic profiles was observed in most MSCs cultures from *S. cerevisiae* TC1517 (Table S5, Figure S2) and YI30 (Table S6, Figure S3). As reported in Figure 4B, within the TC1517 progeny, the greatest variability was focused on the amide (W2) and fatty acid (W1) regions.

Huge variations were observed in TC_9 in the fatty acid, amide, and carbohydrate regions (Figure 4B). Statistical analysis of growth parameters showed significant differences for all the tested MSCs in comparison to the parental strain except TC_9, whose t_max was the only one significantly different (*p* < 0.05) from the parental yeast. TC_23, which displayed a metabolome similar to the parental, was characterized by a t_max statistically divergent from the parental (*p* < 0.01).

Considering YI30, MSCs also showed significant differences in W1 and W2 regions (Figure 4C), with seven out of the twelve selected MSCs carrying metabolomic changes also in the mixed region (W3). No metabolomic alteration was instead detected for the carbohydrate metabolism (W4). The MSC YI_30 shared the metabolome of its parental strain except for a few wavelengths in the W2 region. Of the eleven MSCs exhibiting metabolomic differences in SWA, only four responded differently to the statistical analysis of growth parameters, while YI_16, YI_20, YI_44, and YI_53 showed no significant differences (*p* < 0.01) compared to the parental strain (Table S3).

Overall, FTIR fingerprinting of the tested MSCs clearly indicates a specific progeny signature. The lowest metabolomic changes were detected within the YVGC13A-derived MSC. Conversely, the sporulation of TC1517 and YI30 parental strains pushed the metabolomic variability of MSCs into the amides (W2) region, also triggering a response in the W1 and W3 regions for TC1517 and YI30 MSCs, respectively.

Based on both metabolomic and growth phenotypes, six MSCs for each parental strain were further selected to be representative of the variability produced by sporulation by choosing those with lower, higher, and PS-like growth phenotypes as well as similar or different metabolomic traits.

### 3.4. Growth and Metabolomic Phenotypes under Stressing Conditions

In order to further assess the phenotypic changes due to the genetic reshuffle mediated by sporulation, the second set of selected MSCs were tested for growth and metabolomic changes (Tables S7–S9; Figures S4–S6) once exposed to stressors specific to the parental strain background (Table 1).

The choice of copper as a stressor for the YVGC13A strain, isolated from vine bark in Canada, is based on the evidence that copper-based fungicides have been used in vineyards for more than 100 years and copper sulphate-based fungicides are the only chemicals allowed under organic standards [46]. High glucose concentrations can damage yeast cells and hamper their normal growth and metabolism [47]. The effect of high glucose levels on altering cell metabolism is therefore particularly interesting for a strain such as TC1517 isolated from grape marc [26]. Finally, since *S. cerevisiae* YI30 has been described as a promising candidate for second-generation bioethanol [13,27,48], formic acid was chosen as one of the most toxic weak acids [26,27] generated during the pre-treatment of lignocellulose wastes and their conversion to ethanol [13].

**Figure 5:**
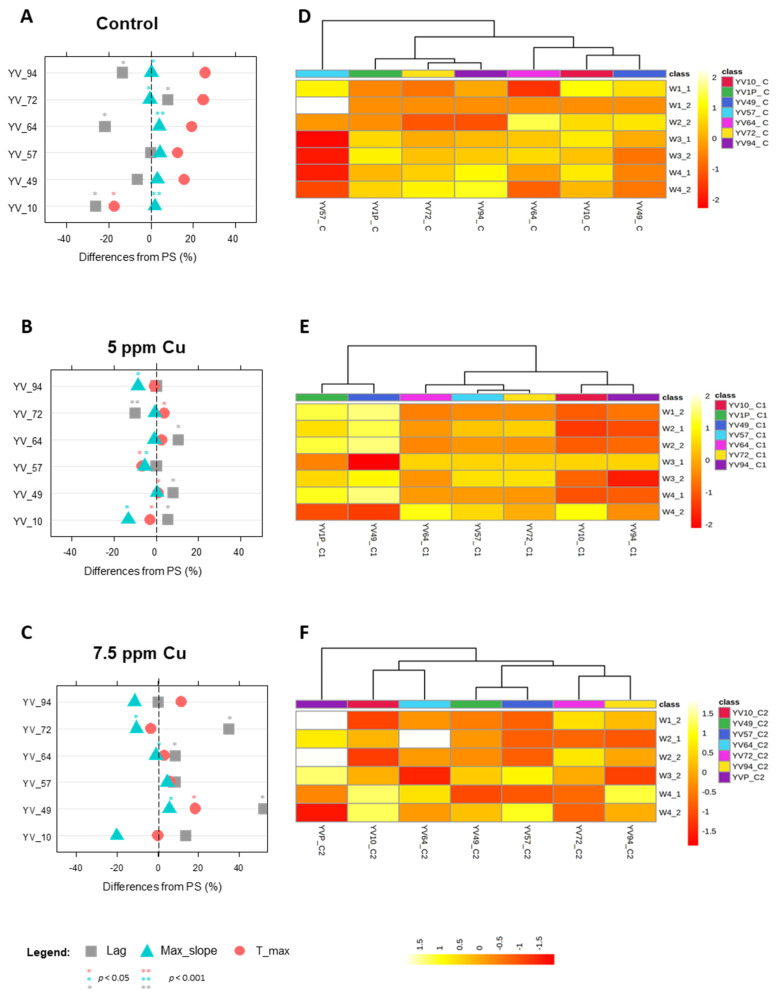
Growth and metabolomic phenotypes of YVGC13A *S. cerevisiae* parental strain and its derived MSCs during growth at increasing concentrations of copper (5–7.5 ppm). Panels (**A**–**C**): Lag time (grey square), max_slope (light blue triangle) and t_max (light red circle) parameters reported as percentage difference respect to the parental strain. Panels (**D**–**F**): Heatmap of the significantly altered FTIR peaks (distance measure using Euclidean, and clustering algorithm using ward.D). The coloured boxes indicate the relative intensities of the mean of peaks in the corresponding spectral region. The colour scale is log2 transformed value and indicates relatively high (yellow) and low (red) peak intensities. Spectral regions have been divided into sub-regions, namely: Fatty acids (W1_1 from 3200 to 3100 cm^−1^–W1_2 from 3098 to 2801 cm^−1^); Amides (W2_1 from 1800 to1649 cm^−1^–W2_2 from 1647 to 1501 cm^−1^); Mixed region (W3_1 from 1499 to 1352 cm^−1^–W3_2 from 1350 to 1202 cm^−1^); Carbohydrates (W4_1 from 1200 to 1053 cm^−1^–W4_2 from 1051 to 902 cm^−1^).

**Figure 6:**
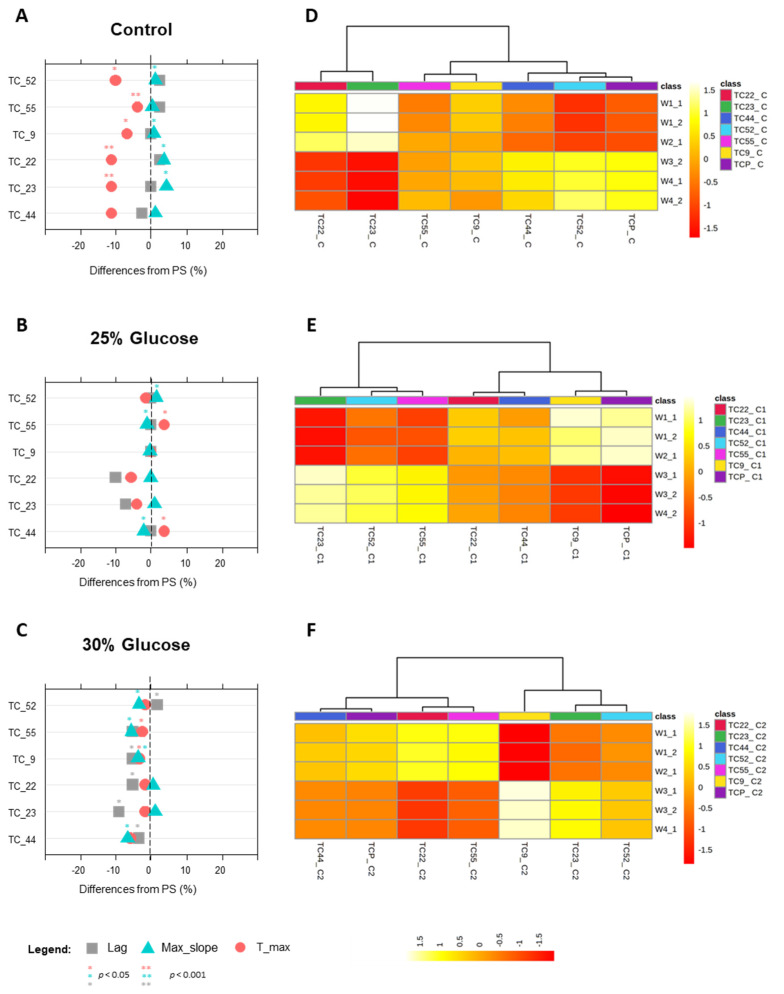
Growth and metabolomic phenotypes of *S. cerevisiae* TC1517 strain and its derived MSCs during growth at increasing concentrations of glucose (25–30%, *w*/*v*). Panels (**A**–**C**): Lag time (grey square), max_slope (light blue triangle) and t_max (light red circle) parameters reported as percentage difference respect to the parental strain. Panels (**D**–**F**): Heatmap of the significantly altered FTIR peaks (distance measure using Euclidean, and clustering algorithm using ward.D). The coloured boxes indicate the relative intensities of the mean of peaks in the corresponding spectral region. The colour scale is log2 transformed value and indicates relatively high (yellow) and low (red) peak intensities. Spectral regions have been divided into sub-regions, namely: Fatty acids (W1_1 from 3200 to 3100 cm^−1^–W1_2 from 3098 to 2801 cm^−1^); Amides (W2_1 from 1800 to1649 cm^−1^–W2_2 from 1647 to 1501 cm^−1^); Mixed region (W3_1 from 1499 to 1352 cm^−1^–W3_2 from 1350 to 1202 cm^−1^); Carbohydrates (W4_1 from 1200 to 1053 cm^−1^–W4_2 from 1051 to 902 cm^−1^).

**Figure 7:**
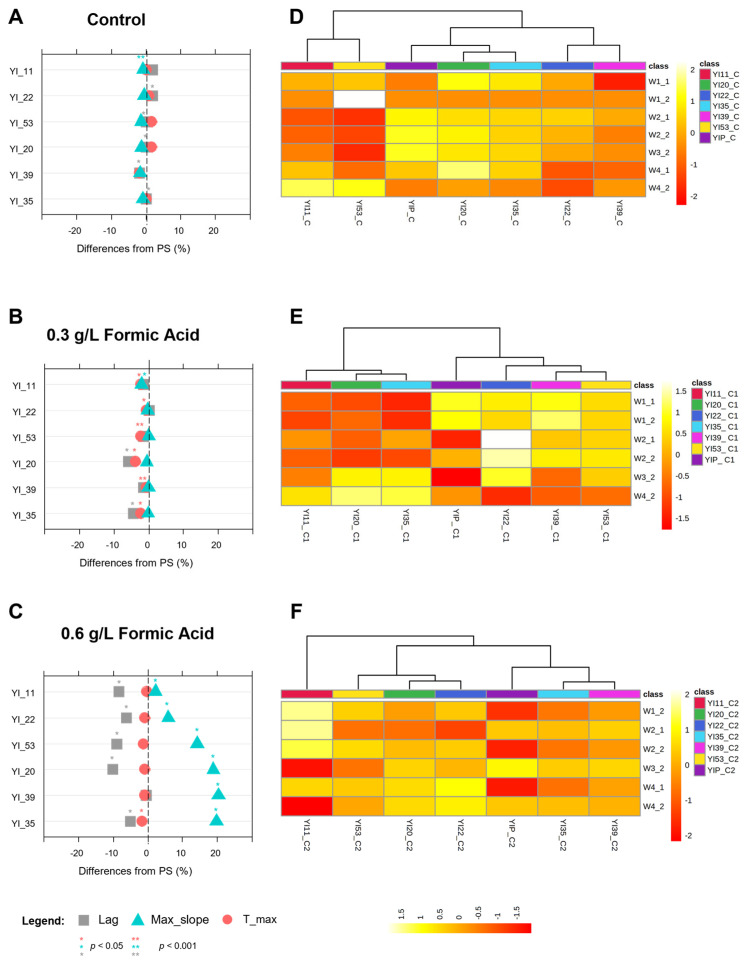
Growth and metabolomic phenotypes of YI30 *S. cerevisiae* parental strain and its derived MSCs during growth at increasing concentrations of formic acid (0.3 and 0.6 g L^−1^). Panels (**A**–**C**): Lag time (grey square), max_slope (light blue triangle) and t_max (light red circle) parameters reported as percentage difference respect to the parental strain. Panels (**D**–**F**): Heatmap of the significantly altered FTIR peaks (distance measure using Euclidean, and clustering algorithm using ward.D). The coloured boxes indicate the relative intensities of the mean of peaks in the corresponding spectral region. The colour scale is log2 transformed value and indicates relatively high (yellow) and low (red) peak intensities. Spectral regions have been divided into sub-regions, namely: Fatty acids (W1_1 from 3200 to 3100 cm^−1^–W1_2 from 3098 to 2801 cm^−1^); Amides (W2_1 from 1800 to1649 cm^−1^–W2_2 from 1647 to 1501 cm^−1^); Mixed region (W3_1 from 1499 to 1352 cm^−1^–W3_2 from 1350 to 1202 cm^−1^); Carbohydrates (W4_1 from 1200 to 1053 cm^−1^–W4_2 from 1051 to 902 cm^−1^).

Overall, when grown under increasing concentrations of stressing agents, both parental strains and related MSCs displayed a dose-dependent response (Figure 5, Figure 6 and Figure 7).

#### 3.4.1. Phenotypes under Copper Sulphate Stress

*S. cerevisiae* YVGC13A-progeny were tested for the ability to withstand increasing concentrations of copper sulphate. Growth performances in the benchmark broth SD (Figure 5A) revealed significant differences in lag and max_slope values (*p* < 0.05) for all MSCs tested, except for YV_49 and YV_57. YV_10, YV_64, and, to a lesser extent, YV_94, showed the most interesting phenotypes for the simultaneous increase in the max_slope and decrease in the lag phase, significantly improving the growth kinetics of these MSCs.

Additionally, differences with respect to the parental strain were even more intense once considering the metabolomic reactions (Figure 5D, Table S7, Figure S4). Although with similar growth kinetics, the YV_57 MSC exhibited a metabolomic pattern more divergent from the PS, downregulating bands in mixed and carbohydrate regions (W3 and W4) and up-regulating those in W1. Conversely, YV_72 and YV_94 MSCs, which showed significantly different lag phases, displayed more similar metabolomic patterns, except for an increase in W4 and a decrease in W2 band intensities. The other three MSCs, YV_10, YV_49, and YV_64, had a pattern slightly reversed from that of PS, reducing W4 and increasing W2.

Once exposed to 5 ppm of copper, most MSCs showed higher sensitivities than *S. cerevisiae* YVGC13A, characterized by a longer lag phase and a lower max_slope (Figure 5B). Only the YV_72 displayed a lag phase shorter than PS and can therefore be considered the most tolerant MSC at this copper concentration. The heatmap of the significantly altered FTIR peaks (Figure 5E) highlighted how the increased sensitivity of the MSCs corresponded to a general decrease in intensity of the whole spectra except for the W4_2 bands in the YV_10, YV_57, YV_64, and YV_72 MSCs. Among all MSCs, only the YV_49 clustered together with the PS.

As the copper concentration increased (7.5 ppm), a quite different scenario was depicted (Figure 5C, Table S7, Figure S4). YV_10, YV_57, and YV_94 MSCs seem to increase their tolerance, by showing the same phenotype as *S. cerevisiae* YVGC13A. Conversely, YV_64 and YV_72 MSCs displayed increased sensitivity, attributable to the longer lag and the reduction of the max_slope values. Noteworthy, the YV_49 displayed higher values than the PS for all growth parameters considered. Despite the longer lag phase, this culture was then able to grow more rapidly during the log phase, giving a higher cell density than the parental strain YVGC13A.

In reaction to this copper concentration, we observed an increase in the variability of metabolomic profiles, resulting in MSC-dependent signatures (Figure 5F). Overall, the metabolomic patterns of MSCs mirrored those of PS, depicting a general slowing of metabolism to the exclusion, in a few cases, of carbohydrates in the W4_2 region.

With respect to its progeny, *S. cerevisiae* YVGC13A reacted to copper’s supplementation by inducing genes responsible for carbohydrates metabolism and protein biosynthesis as a general mechanism of stress response in this species (Figure 5E,F) [45,49]. Conversely, the higher intensities in fatty acids may be the result of higher reactive oxygen species (ROS) accumulation, already described as a specific *S. cerevisiae* response to stress conditions triggered by copper [50,51].

Generally, the sporulation of YVGC13A has produced a significant amount of variability, resulting in some improved phenotypes, both at rest and under stressful conditions. However, the MSCs that performed better in control broth were not the same ones that displayed increased tolerance to copper supplementation. These data supported the hypothesis that the extensive sporulation applied in this study increases the amount of available variability compared to those procedures implying sporulation under selection conditions.

#### 3.4.2. Phenotypes under Glucose Stress

In the case of TC1517-derived progeny, the control in SD broth was useful to confirm and further investigate the differential behaviors of the selected MSCs both in terms of growth parameters (Figure 6A) and alteration of cell metabolomes (Figure 6D).

Only the TC_44 displayed growth parameters such as those of the parents. The other MSCs were able to grow faster than the parental strain, with significantly higher t_max and max_slope values (Figure 6A). Remarkably, the increase in the growth rate of TC_22 and TC_23 was linked to specific metabolomic changes induced by the up-regulation of fatty acids and amides bands (W1 and W2_1) coupled with the down-regulation of mixed and carbohydrates ones (W3_2 and W4) (Table S8, Figure S5). The fingerprints of these MSCs were reversed with respect to those of *S. cerevisiae* TC1517 (Figure 6D). The other four MSCs displayed lower changes and were mainly located in the W1 and W2_1 regions.

At increasing glucose concentrations, significant alterations were evident in both the growth and metabolomic phenotypes of the selected MSCs. In the presence of 25% glucose, TC_52 was the only MSC that significantly increased the growth rate (max_slope) compared to that of the parental, as already revealed in the control condition. No significant differences were detected for the other MSCs, except for TC_44 and TC_55, both affected by a significant increase in t-max values and a reduction in max slope values (*p* < 0.05), resulting in a reduced growth rate (Figure 6B).

Furthermore, once challenged with 25% glucose, the parental strain expressed metabolomic changes opposite those observed in the benchmark broth (Figure 6E). The increase in glucose concentration prompted higher intensities of fatty acid and protein bands (W1 and W2_1) together with a reduction of those in mixed and carbohydrate regions (W3_2 and W4). The same response was observed for TC_9 MSC, which, noteworthily, exhibited growth performances such as those of *S. cerevisiae* TC1517. On the contrary, TC_23, TC_52, and TC_55 MSCs had an antithetical regulation in these spectral regions. Additionally, the last two MSCs, sharing similar metabolomic footprints, showed opposite growth behaviors. Finally, the TC_44 exhibited band intensities around neutrality for all spectral regions.

The glucose supplementation up to 30% significantly affected the growth parameters (*p* < 0.05) and led to a general reduction of all MSCs’ growth (Figure 6C). In TC_9, growth reduction was accompanied by a substantial alteration of cell metabolism with the down-regulation of fatty acids (W1) and proteins (W2) and the up-regulation of mixed (W3) and carbohydrate (W4) regions (Figure 6F). A similar response, though of lesser intensity, was displayed by TC_23 and TC_52 MSCs. The TC_22 and TC_55 clustered separately because of the opposite response to that of these three MSCs. At glucose 30%, TC_44 was the only MSC that displayed the same metabolomic alteration as the parental TC1517.

Overall, the sporulation of TC1517 has triggered a renewed level of variability that has impacted both the growth and metabolomic phenotypes of the six MSCs selected. In the absence of glucose stress, the growth phenotypes of most spores improved compared to the parental strain. Conversely, high glucose levels induced a general worsening of growth performance by reducing the growth rate of the MSCs. The only exception was the TC_52 culture, which maintained a growth rate higher than that of the parental cells even under 25% glucose.

Interestingly, TC_22 and TC_23 MSCs, which demonstrated the best performance under control conditions, reacted to glucose addition by significantly shaping their metabolism in the direction of a reduction of the lag phase, a typical response of strains with increased tolerance to a specific stressor [52,53].

The metabolomic fingerprint of these strains, despite having a dose-dependent pattern, showed a peculiarity in the constant clustering of the responses of W1 and W2 in opposition to those of W3 and W4, both under control and in stressed conditions. This evidence could be attributed mainly to the opposite regulation of genes involved in protein and carbohydrate metabolism. The fact that some stressing conditions induce carbohydrate metabolism genes by down-regulating those involved in protein biosynthesis has already been observed in *S. cerevisiae* [54]. It is well documented that *S. cerevisiae* cells accumulate some carbohydrates in response to different types of stress [45,49,55]. In the presence of 15 g L^−1^ of glucose, the production of intracellular glycerol and trehalose was found to be significantly increased [47]. Furthermore, glucose concentration has been reported to have a proportional effect on intracellular ROS, which increases intensity in the W1_1 region [56].

#### 3.4.3. Phenotypes under Formic Acid Stress

When grown in control broth, most of the MSCs of *S. cerevisiae* YI30 were affected by a significant increase in the lag phase (*p* < 0.05) with respect to the parental strain (Figure 7A). The variability induced by sporulation also interested the max slope value in YI_11, which exhibited the worst phenotype together with YI_22.

Based on the metabolomic alterations (Table S9, Figure S6), MCSs were grouped into two main clusters (Figure 7D). The first included four MSCs, of which YI_20 and YI_35 are closest to the PS, whereas YI_22 and YI_39 are in a separate subcluster characterized by a general downregulation of bands in all spectral regions. The second group consisted of YI_11 and YI_53 MSC, which were separated from the PS mainly by reduced intensities in the amide bands and increased signals for carbohydrates.

The phenotypes described for growth in a resting condition significantly changed in response to formic acid, according to the increase in dose (Figure 7B,C). The presence of 0.3 g L^−1^ formic acid modified growth phenotypes except for YI_11, which maintained the same pattern displayed in the control broth (Figure 7B). No significant differences from the parental strain were detected for YI_22, YI_39, and YI_53, while YI_20 and YI_35 were faster thanks to the significant reduction in Lag and t_max (*p* < 0.05).

At the highest tested concentration (0.6 g L^−1^), formic acid clearly prompted the growth of all MSCs by reducing the lag parameter with respect to the PS (*p* < 0.05), with the only exception of YI_39 MSC (Figure 7C).

The heatmaps of the significantly altered FTIR peaks (Figure 7E,F) showed that the differential ability to withstand formic acid was mediated by a fine tuning of carbohydrates, proteins, and fatty acid pathways. In addition, the improved performance of YI_20 and YI_35 in the presence of 0.3 g L^−1^ formic acid (Figure 7E), supported by the strong down-regulation in W1 and up-regulation in W3_2 and W4_2 regions, could be explained considering that *S. cerevisiae,* under some stressful conditions, induced genes involved in carbohydrate metabolism while down-regulating those involved in protein biosynthesis. In addition, the metabolomic pattern shown by YI_11 at higher concentrations (Figure 7F) suggests that other mechanisms may be involved in the response to formic acid stress. ROS, which are potentially responsible for providing tolerance to toxic formic acid, fall in the fatty acid region (W1_1). The higher intensity in W1_1 bands displayed by YI_11 can possibly be related to the higher accumulation of ROS [56]. This hypothesis is under investigation using a focused LC/MS approach.

Overall, the sporulation of YI30 resulted in MSCs with differential ability to withstand increasing concentrations of formic acid and was useful for the selection of a few candidates with promising phenotypes to be further studied to both shed light on the still poorly investigated mechanism of formic acid tolerance in *S. cerevisiae* and to develop superior yeast strains with increased resistance to this weak acid [57,58]. Few MSCs, indeed, showed a lower lag phase, thus reacting much faster than the parental strains thanks to strong and strain-specific intracellular metabolomic reactions.

## 4. Conclusions

The main hypothesis of this paper is that the proposed non-GMO approach was efficient in renewing genetic variability through the extensive sporulation of three *S. cerevisiae* strains with different origins and backgrounds. The procedure involved an initial randomized sampling of the MSCs produced by the extensive sporulation of each strain, without any preliminary selection. In addition, a series of sequential steps focused on the analysis of growth performances and metabolomic reactions allowed the analysis to be restricted to six MSCs for each strain, screened at rest and under specific stress conditions. Overall, data confirmed that *i.* the genome renewal reintroduced a quote of variability, selectable following the approach presented in this study, *ii.* the extensive sporulation generates variability in both growth and metabolomic phenotypes; and *iii.* this variability depends on the starting parental strain, proving that the geographical location and ecological origin of yeast have a major signature on its phenotypic pattern. Although the ongoing whole genome sequencing of selected MSCs will clarify the nature and stability of this variability, this novel procedure looks very promising for renewing yeast genetic variability as a tool to obtain improved organisms with specific phenotypes and industrial fitness. Further, selected MSCs are indeed of great metabolomic interest towards the identification of molecules with deep impact on the yeast resistome against specific stressors.

## Figures and Tables

**Table 1:** Strains used in this study: background, origin and tested stressing agents.

Strain	Background	Geographical Location	Genotype	Reference	Stressing Agent	Low Stress	High Stress
TC1517	Grape marcs	Italy	2n, homotallic	[26]	Glucose (g L^−1^)	250	300
YI30	Industrial distillery	South Africa	2n, homotallic	[27]	Formic acid (g L^−1^)	0.3	0.6
YVGC13A	Vineyard, isolated from vine bark	Canada	2n, homotallic	University of Perugia	Copper sulfate (Cu-ppm)	5	7.5

## Data Availability

Not applicable.

## Supplementary Materials

Table S1: The following supporting information can be downloaded at: https://www.mdpi.com/article/10.3390/jof9020264/s1, Probability associated with the Student’s *t*-test (*p*-value) of all MSC compared to the parental strain (YVGC13A), Table S2: The probability associated with the Student’s *t*-test (*p*-value) of all MSC compared to the parental strain (TC1517); Table S3: Probability associated with the Student’s *t*-test (*p*-value) of all MSC compared to the parental strain (YI30); Table S4: FTIR absorbance spectra of the YVGC13A strain recorded at the end of the exponential phase of growth in SD broth supplemented with 2% glucose, Table S5: FTIR absorbance spectra of the TC1517 strain recorded at the end of the exponential phase of growth in SD broth supplemented with 2% glucose, Table S6: FTIR absorbance spectra of the YI30 strain recorded at the end of the exponential phase of growth in SD broth supplemented with 2% glucose, Table S7: FTIR absorbance spectra of theYVGC13A strain under copper sulphate supplementation; Table S8: FTIR absorbance spectra of the TC1517 strain under glucose supplementation; Table S9: FTIR absorbance spectra of YI30 strain under formic acid supplementation, Figure S1: FTIR absorbance spectra of YVGC13A strain recorded at the end of the exponential phase of growth in SD broth supplemented with 2% glucose, Figure S2: FTIR absorbance spectra of TC1517 strain recorded at the end of the exponential phase of growth in SD broth supplemented with 2% glucose, Figure S3: FTIR absorbance spectra of YI30 strain recorded at the end of the exponential phase of growth in SD broth supplemented with 2% glucose, Figure S4: FTIR absorbance spectra of YVGC13A strain under copper sulphate supplementation, Figure S5: FTIR absorbance spectra of TC1517 strain under glucose supplementation, Figure S6: FTIR absorbance spectra of YI30 strain under formic acid supplementation.
