# Supplementary material for: Renewing Lost Genetic Variability with a Classical Yeast Genetics Approach
Source: J Fungi (Basel). 2023 Feb 16;9(2):264. doi: 10.3390/jof9020264 (PMC9958831; doi:10.3390/jof9020264)
Supplement: Supplementary file 1 [file jof-09-00264-s001.zip › Supplementary tables_t-Test-F.pdf]

Table S1: Probability associated with the Student's *t*-Test (*p*-value) of all monosporal colonies compared to the parental strain (YVGC13A).

MSCs	lag	t_max	max_slope
YV_1	0.000	0.017	0.174
YV_2	0.002	0.008	0.054
YV_3	0.018	0.041	0.047
**YV_10**	0.047	0.275	0.005
**YV_17**	0.005	0.017	0.431
**YV_19**	0.003	0.002	0.084
YV_25	0.005	0.041	0.002
YV_28	0.008	0.006	0.486
**YV_29**	0.012	0.035	0.309
YV_35	0.032	0.097	0.217
YV_36	0.019	0.074	0.087
**YV_37**	0.048	0.107	0.357
YV_47	0.003	0.017	0.340
YV_48	0.019	0.067	0.175
**YV_49**	0.029	0.005	0.261
**YV_55**	0.032	0.017	0.254
YV_56	0.032	0.222	0.026
**YV_57**	0.019	0.333	0.041
YV_63	0.019	0.330	0.062
**YV_64**	0.001	0.013	0.090
**YV_65**	0.005	0.001	0.124
**YV_72**	0.019	0.191	0.041
YV_73	0.059	0.064	0.439
YV_74	0.010	0.104	0.203
YV_82	0.002	0.006	0.398
YV_83	0.007	0.024	0.022
YV_84	0.000	0.005	0.107
YV_93	0.010	0.005	0.009
**YV_94**	0.000	0.029	0.012
YV_95	0.004	0.037	0.005

Legend: Student's *t*-test was performed to compare growth parameters (lag, t_max, max_slope) of MSCs with the parental strain. The monosporal colonies selected for further analysis are highlighted in bold.

Table S2: Probability associated with the Student's *t*-Test (*p*-value) of all monosporal colonies compared to the parental strain (TC1517).

MSCs	lag	t_max	max_slope
TC_1	0.500	0.104	0.262
TC_2	0.047	0.035	0.435
TC_3	0.036	0.000	0.410
TC_7	0.371	0.037	0.288
TC_8	0.018	0.003	0.371
**TC_9**	0.065	0.017	0.201
TC_12	0.092	0.009	0.146
TC_13	0.019	0.001	0.317
**TC_14**	0.019	0.004	0.028
TC_17	0.010	0.004	0.244
TC_19	0.004	0.004	0.137
TC_20	0.000	0.001	0.303
TC_21	0.005	0.001	0.496
**TC_22**	0.017	0.003	0.456
**TC_23**	0.018	0.001	0.293
**TC_25**	0.004	0.001	0.076
TC_29	0.059	0.018	0.057
TC_30	0.092	0.007	0.221
TC_31	0.027	0.007	0.029
**TC_33**	0.003	0.000	0.053
TC_37	0.008	0.001	0.221
TC_38	0.007	0.000	0.075
TC_39	0.004	0.001	0.251
TC_41	0.016	0.006	0.122
**TC_42**	0.019	0.002	0.165
**TC_44**	0.010	0.002	0.134
**TC_48**	0.011	0.004	0.094
**TC_49**	0.003	0.001	0.049
**TC_52**	0.002	0.002	0.095
**TC_55**	0.010	0.003	0.100

Legend: Student's *t*-test was performed to compare growth parameters (lag, t_max, max_slope) of MSCs with the parental strain. The monosporal colonies selected for further analysis are highlighted in bold.

Table S3: Probability associated with the Student's t-Test (*p*-value) of all monosporal colonies compared to the parental strain (YI30).

MSCs	lag	t_max	max_slope
YI_9	0.018	0.047	0.031
YI_10	0.000	0.003	0.090
**YI_11**	0.018	0.004	0.070
**YI_16**	0.019	0.019	0.384
YI_18	0.029	0.005	0.206
YI_19	0.018	0.004	0.057
**YI_20**	0.000	0.018	0.029
**YI_22**	0.008	0.000	0.070
**YI_30**	0.005	0.005	0.031
**YI_35**	0.010	0.003	0.098
**YI_35**	0.059	0.127	0.003
**YI_39**	0.000	0.264	0.002
**YI_42**	0.005	0.032	0.495
**YI_44**	0.037	0.019	0.147
**YI_47**	0.036	0.004	0.148
**YI_53**	0.018	0.018	0.059
YI_55	0.029	0.008	0.049
**YI_56**	0.010	0.069	0.002
YI_57	0.005	0.005	0.023
YI_66	0.029	0.029	0.010
YI_67	0.005	0.004	0.018
YI_70	0.029	0.008	0.059
YI_76	0.037	0.037	0.073
YI_78	0.011	0.011	0.060
YI_80	0.029	0.004	0.021
YI_85	0.029	0.036	0.021
YI_86	0.029	0.004	0.020
YI_87	0.048	0.005	0.021
YI_102	0.018	0.004	0.029
YI_103	0.065	0.019	0.069

Legend: Student's *t*-test was performed to compare growth parameters (lag, t_max, max_slope) of MSCs with the parental strain. The monosporal colonies selected for further analysis are highlighted in bold.
